# Perivascular Adipose Tissue Becomes Pro-Contractile and Remodels in an IL10^−/−^ Colitis Model of Inflammatory Bowel Disease

**DOI:** 10.3390/ijms251910726

**Published:** 2024-10-05

**Authors:** Samuel W. Jenkins, Elizabeth A. Grunz, Kassandra R. Ramos, Erika M. Boerman

**Affiliations:** Department of Medical Pharmacology and Physiology, University of Missouri, Columbia, MO 65212, USA

**Keywords:** perivascular adipose tissue, vascular inflammation, inflammatory bowel disease, mesenteric arteries, colitis

## Abstract

Inflammatory Bowel Diseases (IBDs) are associated with aberrant immune function, widespread inflammation, and altered intestinal blood flow. Perivascular adipose tissue (PVAT) surrounding the mesenteric vasculature can modulate vascular function and control the local immune cell population, but its structure and function have never been investigated in IBD. We used an IL10^−/−^ mouse model of colitis that shares features with human IBD to test the hypothesis that IBD is associated with (1) impaired ability of PVAT to dilate mesenteric arteries and (2) changes in PVAT resident adipocyte and immune cell populations. Pressure myography and electrical field stimulation of isolated mesenteric arteries show that PVAT not only loses its anti-contractile effect but becomes pro-contractile in IBD. Quantitative immunohistochemistry and confocal imaging studies found significant adipocyte hyperplasia and increased PVAT leukocytes, particularly macrophages, in IBD. PCR arrays suggest that these changes occur alongside the altered cytokine and chemokine gene expression associated with altered NF-κB signaling. Collectively, these results show that the accumulation of macrophages in PVAT during IBD pathogenesis may lead to local inflammation, which ultimately contributes to increased arterial constriction and decreased intestinal blood flow with IBD.

## 1. Introduction

Inflammatory bowel diseases (IBDs), including Crohn’s disease and ulcerative colitis, are chronic autoimmune diseases with significant morbidity and a rapidly rising worldwide incidence [1] of ~1.3% of the adult population in the United States [2]. All IBD cases are associated with significant intestinal inflammation, and many present with significant extraintestinal manifestations including a wide range of cardiovascular conditions. In the vasculature, IBD is associated with a poor regulation of blood flow with inflamed regions experiencing increased flow through the microcirculation even as the entire intestine is underperfused by larger resistance vessels [3,4]. Further, increased intestinal blood flow is a predictor of treatment success [5], and decreased flow is considered an early marker of relapse [6]. Thus, understanding whether and how different vascular beds are affected by IBD is important for predicting and improving clinical outcomes. The overall goal of our research is to understand how arteries are impacted by IBD and identify potential mechanisms to preserve blood flow during disease pathogenesis.

In the limited studies of the vasomotor function in IBD, decreased vasodilatory capacity across multiple mechanisms appears to be an important mediator of vascular dysfunction. In the intestinal microcirculation, endothelial dysfunction appears important, as flow-mediated dilation is decreased [7] and nitric oxide-dependent dilation is impaired and replaced by cyclooxygenase mechanisms [8]. In elastic arteries, stiffening appears to be a key factor based on increased pulse wave velocity in carotid and femoral arteries [9,10]. There is also a strong correlation between arterial stiffness and markers of chronic inflammatory burden [11], highlighting mechanistic links between IBD and atherosclerotic disease risk. Surprisingly, few studies have directly investigated the impact of IBD on the function of the mesenteric arteries that regulate blood flow to the intestines. Our work on vasomotor function in the mesenteric resistance arteries previously demonstrated an important role for the adventitia in vascular dysfunction. In our mouse model, perivascular sensory nerve-mediated dilation is nearly abolished with IBD [12]. In parallel, macrophages accumulate in the adventitia, and their depletion with clodronate liposomes restores sensory vasodilation [13], highlighting a role for both nerves and immune cells in IBD-related vasomotor dysfunction. These findings further suggest that it may be important to investigate the role of a second nerve containing and immune-cell rich vascular tunica capable of modulating vascular function—the perivascular adipose tissue.

To date, no studies of resistance vessel function in IBD have addressed the structural or functional effects of IBD on perivascular adipose tissue (PVAT). PVAT surrounds nearly all pre-capillary blood vessels, and healthy PVAT exerts a net anti-contractile effect on underlying arteries through the complex secretome of its dynamic populations of adipocytes, leukocytes and nerves. PVAT loses its anti-contractile function in many disease states, including the cardiovascular diseases comorbid with IBD [14]. Adipose dysfunction across fat depots, including PVAT, is linked to the development of cardiovascular complications in autoimmune diseases. As outlined in detail in a recent review [15], clinical studies of patients with rheumatic autoimmune diseases such as rheumatoid arthritis, psoriasis and vasculitis point to PVAT inflammation and remodeling as a driving factor for the pathogenesis of cardiovascular disease in those patients. In Crohn’s disease, an inflammatory, fibrotic adipose depot known as “creeping fat” wraps around the intestine, contributing to stricture, inflammation and increased disease severity [16,17]. Based on these lines of evidence and our previous work in the mesenteric artery adventitia, we sought to determine whether PVAT contributes to vascular dysfunction in IBD. While no current mouse models exactly recapitulate all clinical features of human IBD, several accepted colitis models, including the IL-10^−/−^ mouse model used here, have emerged as useful tools that share many physiological characteristics of human IBD, including histological colon inflammation, immune mediation of colitis, and involvement of the microbiome in colitis development [18,19,20]. This study uses pressure myography, histological analysis, confocal immunolabeling and gene expression arrays to test the hypotheses that PVAT contributes to hypoperfusion in IBD by the loss of anti-contractile function in association with structural remodeling and an increase in the local macrophage population.

## 2. Results

### 2.1. PVAT Becomes Pro-Contractile in Mouse Mesenteric Arteries with IBD

**Figure 1:**
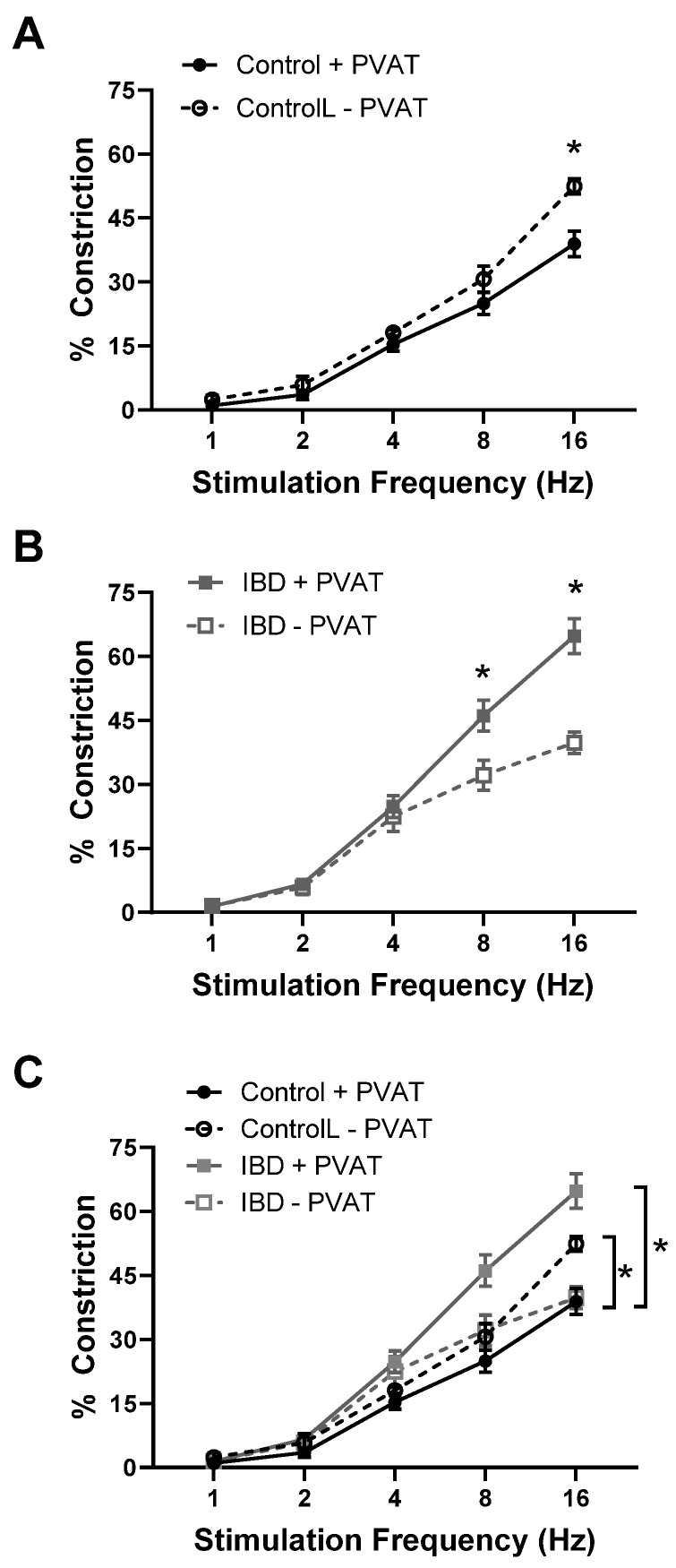
PVAT becomes pro-contractile in mesenteric arteries with inflammatory bowel disease (IBD). Data are mean ± SE for % constrictions to electrical field stimulation (EFS, 1–16 Hz) of cannulated, pressurized mesenteric arteries with (+PVAT) and without (−PVAT) perivascular adipose tissue (PVAT) from Control and IBD mice. PVAT presence reduces electrical field stimulation (EFS)-induced constriction in Control arteries (**A**,**C**) but increases constriction in IBD arteries (**B**,**C**). *n* = 4–6 per group, * = *p* < 0.05 for +PVAT vs. −PVAT (**A**,**B**) or Control vs. IBD (**C**).

Pressure myography and the electrical field stimulation of mesenteric arteries with and without PVAT (+PVAT and −PVAT) were used to determine the impact of PVAT presence on vasoconstriction. Consistent with previous studies [13,21], IBD was associated with a ~25% decrease in maximum constriction in IBD vs. Control arteries -PVAT (Figure 1A–C). In control arteries, the presence of PVAT significantly decreased constriction with maximum observed constrictions reduced by ~25% (Figure 1A,C). In contrast, the presence of PVAT in arteries from IBD significantly increased vasoconstriction with maximum constrictions increased over 50% (Figure 1B,C). These data highlight a switch from anti-contractile PVAT in healthy arteries to pro-contractile PVAT in IBD.

### 2.2. PVAT Remodels with IBD

**Figure 2:**
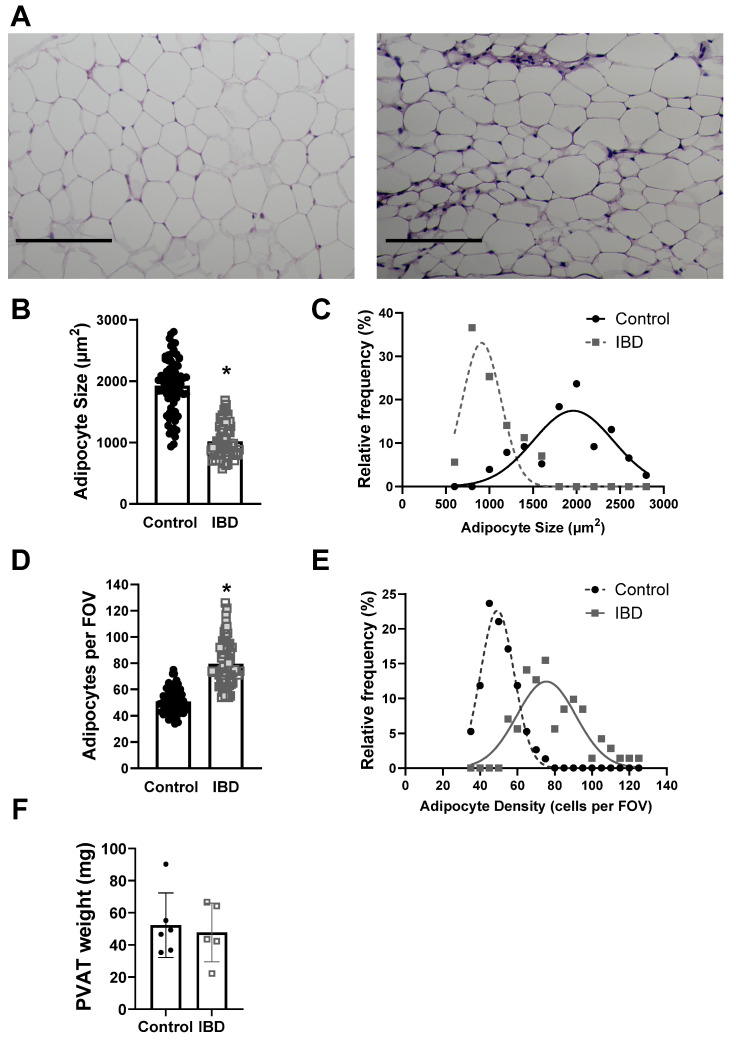
IBD cases PVAT adipocyte hyperplasia. (**A**) Representative imaged of H&E-stained sections of mesenteric artery PVAT from Control (**left**) and IBD (**right**) arteries. Scale bar = 100 µm. Quantitative analysis of H&E images showed IBD-associated decreases in mean adipocyte size (**B**) but an increase in size distribution (**C**), mean adipocyte density (**D**) and density distribution (**E**). *n* = 71 (Control) or 76 (IBD) images analyzed from 16 PVAT samples each with 4 mice per group. * = *p* < 0.05 via nested *t*-test. Total mesenteric PVAT wet weight was not different in IBD vs. Control mice (**F**).

To determine whether structural changes to PVAT adipocytes occur alongside functional alterations in IBD, the adipocyte size and density were measured in H&E stained PVAT sections from Control and IBD mice. Adipocytes were larger in Control vs. IBD (1926 ± 51 vs. 1018 ± 32 μm^2^, Figure 2A,B) with decreased density (50.7 ± 1.0 vs. 79.4 ± 2.0 cells/field, Figure 2A,D), suggesting adipocyte hyperplasia. Frequency histograms for size and density show that IBD is also associated with an increase in the distribution of size and density (Figure 2C,E). IBD did not alter total PVAT weight (Figure 2F) or body weight, suggesting changes are not simply due to a decrease in the amount of PVAT present around mesenteric arteries. IBD was also associated with an increase in stromal vascular fraction, as shown by increased purple staining (Figure 2A), suggesting an increase in non-adipocyte cells in PVAT with IBD.

### 2.3. Macrophages Accumulate in PVAT with IBD

**Figure 3:**
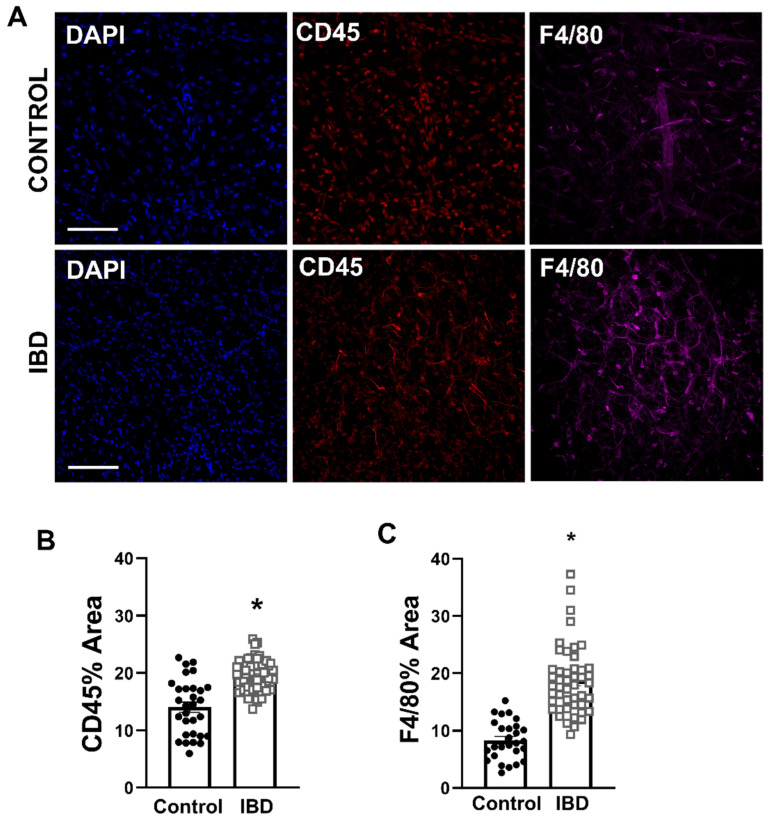
Macrophages accumulate in mesenteric artery PVAT with IBD. (**A**) Representative confocal z-projections of PVAT labeled for nuclei (DAPI, blue, **left**), leukocytes (CD45, red, **center**) and macrophages (F4/80, magenta, **right**) from Control (**top**) and IBD (**bottom**) mice. Scale bar = 100 µm. (**B**,**C**) Data are mean ± SE for percent fluorescent area of CD45 (**B**) and F4/80 (**C**). *n* = 27 images from 4 PVAT samples (Control) or 49 (IBD) images from 8 PVAT samples collected from 4 mice per group. * = *p* < 0.05 in Control vs. IBD via nested *t*-test.

Because stromal vascular fraction appeared greater in IBD PVAT and previous work showed an increase in adventitial macrophages with IBD, mesenteric artery PVAT from Control and IBD was immunolabeled for leukocytes (CD45) and macrophages (F4/80) and confocally imaged (Figure 3A). Analysis of the fluorescent images showed that CD45 fluorescence was increased by >50% (Figure 3B), and F4/80 fluorescence was increased by >100% (Figure 3C) in IBD vs. Control samples, suggesting that IBD leads to an influx of leukocytes, particularly macrophages, into mesenteric artery PVAT.

### 2.4. IBD Leads to Altered PVAT Expression of Cytokine and Chemokine Genes

**Figure 4:**
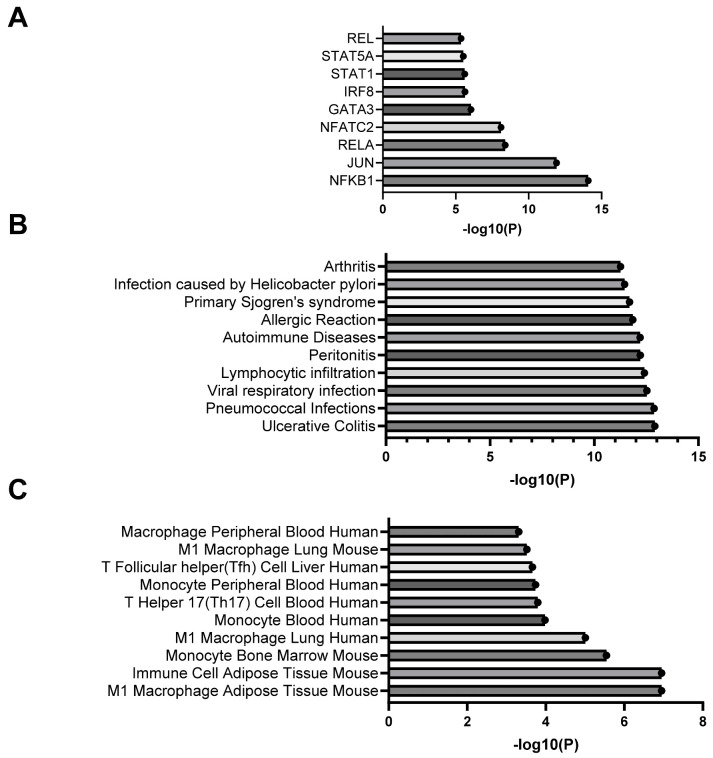
Enrichment analysis of differentially expressed genes from cytokine and chemokine PCR arrays. Data are the top transcription factors (**A**), disease associations (**B**) and cell type associations (**C**) based on analysis of PCR array genes with fold changes > 1.5, <0.5 or *p* < 0.05.

To investigate whether PVAT macrophage accumulation and pro-contractility occurs alongside an increase in cytokine and chemokine gene expression, PCR arrays were used to test for changes in 84 key cytokine and chemokine genes from Control and IBD mesenteric artery PVAT. Ten genes were differentially expressed in IBD vs. Control samples (*p* < 0.05, Table 1 and Table 2). Five genes had significantly increased expression in IBD PVAT: Lymphotoxin B, CCL12, CXCL13, CCL5 and CXCL16 (Table 1), and four genes had significantly decreased expression in IBD PVAT: colony-stimulating factor 3, CCL3, thrombopoetin and colony-stimulating factor 1 (Table 2). We used Enrichr to investigate relevant transcription factors (Figure 4A), disease associations (Figure 4B) and cell-type associations (Figure 4C) based on array genes with IBD PVAT fold-change expressions > 1.5. The transcription factor pathway most enriched was NF-κB (Figure 4A). Consistent with our focus and model, the top disease association was ulcerative colitis (Figure 4B), and the top cell type association was mouse M1 adipose macrophages. Enrichment analysis needs to be interpreted considering only 84 cytokine and chemokine genes were considered in the PCR arrays rather than the entire genome. Overall, PCR arrays highlight significantly altered cytokine and chemokine gene expression in PVAT with IBD with results suggesting the activation of inflammatory signaling.

## 3. Discussion

This is the first study to show in any species or vascular bed that PVAT (1) loses its anti-contractile function and (2) becomes pro-contractile with the development of IBD (Figure 1). This finding is consistent with studies showing a loss of PVAT anti-contractility in obesity [22,23], hypertension [24] and aging [25], suggesting that common mechanisms may exist between diseases. Elucidating such mechanisms is difficult, as PVAT’s role in modulating vasomotor function varies based on a complex and incompletely defined balance of factors including immune cell populations, adipokine and cytokine release profiles, ROS production, neurotransmitter release, signaling gases such as NO and H_2_S, local oxygen concentration, extracellular matrix composition and more. Any direct comparison between published studies and the present work is also complicated by vascular bed differences. The majority of published studies on PVAT’s pro- vs. anti-contractile function are conducted in the abdominal aorta, which is a non-resistance artery with PVAT containing largely brown adipocytes [26]. We focus here on mesenteric resistance arteries with PVAT comprising white adipose tissue based on their role in supplying the intestine that is directly affected by IBD. Throughout the body, the structural plasticity of white adipose allows more rapid changes in adipocyte size and/or number (i.e., hypertrophy vs. hyperplasia) compared to the brown adipocytes [27]. Thus, we first focused on “structural” changes in PVAT with IBD. The hypertrophy (increased cell size) of PVAT white adipose is documented in obesity [28], but IBD-related changes in PVAT were unknown. We found that IBD was associated with PVAT adipocyte hyperplasia (Figure 2) with smaller, more densely populated adipocytes that maintain total PVAT mass even as body weight decreases. Adipocyte hyperplasia is consistent with inflammatory “creeping fat” that wraps around the intestine in human IBD [29], and it makes sense physiologically that the nearby mesenteric PVAT would remodel in similar ways.

The analysis of PVAT H&E sections further suggested an expansion of the stromal vascular fraction with IBD, and we confirmed an increase in CD45+ leukocytes and F4/80+ macrophages in PVAT via the quantitative analysis of fluorescent immunolabeling (Figure 3). Again, this is consistent with findings in the creeping fat of human IBD patients that has an expanded immune cell population, including M1 and M2 macrophages [30]. Results are also consistent with our previous findings of increased macrophages in the adventitial wall of mesenteric arteries with IBD, which were linked to impaired sensory nerve-mediated vasodilation [13]. Outside of IBD, changes in the immune cell populations of PVAT have been documented across a number of diseases. Focusing on macrophages, increases have been demonstrated in mesenteric PVAT in obesity [31] and hypertension [32]. Macrophage expansion in aortic PVAT is also well documented in atherosclerosis [33], which is a known IBD comorbidity. The present data in light of published work suggest, albeit not definitively, that macrophage accumulation in mesenteric PVAT with IBD is linked to the loss of anti-contractility. Additional future studies further characterizing macrophage subpopulations and investigating the vasomotor consequences of macrophage depletion are needed to define more concrete mechanistic links.

In all cases of immune cell expansion in PVAT, a likely cause is the increased production of cytokines and chemokines by the collective PVAT cell population. Mouse cytokine and chemokine PCR arrays of mesenteric PVAT testing found 16/84 target genes had meaningfully higher (fold change > 2, Table 1) or lower (fold change < 0.5, Table 2) expression in IBD vs. Control PVAT. Upregulated in IBD, *ccl12*, *ccl5*, *cxcl13*, *cxcl1*, *cxcl16*, *lta* and *ltb* can all promote the recruitment and/or activation of inflammatory cells including monocytes and macrophages in multiple tissue beds. This can lead to chronic inflammation, which can impair vascular function, ultimately reducing blood flow. Of these genes, *ccl5* (commonly referred to as RANTES) has the most known connections to both PVAT and IBD. As summarized in detail by Nosalski and Guzik [34], RANTES receptor activation in PVAT promotes macrophage recruitment, inflammation and PVAT dysfunction across a number of vascular diseases. Similarly, the increased gene and protein expression of CCL5 is linked to intestinal leukocyte recruitment chronic colitis in both humans and mice [35]. CCL12, a CCR2 ligand and murine analog of macrophage chemoattractant protein 1, is elevated in the PVAT of hypertensive mice [36] and is a known M1 macrophage marker associated with monocyte-derived macrophage recruitment during chronic inflammation [37]. CCL12 has also been suggested as a target to treat colitis in mice [38]. Lymphotoxins, particularly Lymphotoxin A, are linked to the direct inhibition of adiponectin expression in epididymal adipose following myocardial ischemia [39]. If the same is true in PVAT, there may be key implications for vasomotor function, as adiponectin is widely considered a key contributor to PVAT anti-contractile and anti-inflammatory functions [40].

While several cytokines and chemokines associated with increased inflammation, vascular dysfunction and colitis were upregulated in the PCR arrays, the downregulated genes were generally associated with anti-inflammatory activities and tissue repair. Genes downregulated in PVAT with IBD included those for IL-13 (*il13*), IL-24 (*il24*) and IL-22 (*il22*). Each of these cytokines is linked to increased anti-inflammatory M2 macrophage polarization [41,42,43]. Based on these findings and the increased expression of several inflammatory cytokines in our PCR arrays, we expect that the increase in PVAT macrophages here results from a disproportionate increase in M1 rather than M2 macrophages, which are similar to our previous findings in the arterial adventitia during IBD [13]. Contrary to our expectations, the expressions of both *csf1* (M-CSF) and *csf3* (G-CSF) were downregulated in PVAT with IBD. Colony-stimulating factors, particularly M-CSF, are linked to both macrophage recruitment and white adipose hyperplasia [44]. The decreased expression in this study suggests that colony-stimulating factor is not a key player in either macrophage recruitment or adipose remodeling in mesenteric PVAT with IBD. Another unexpected finding was the decreased expression of *ccl3* in PVAT with IBD. The increased gene and protein expression of CCL3 (macrophage inflammatory protein 1 alpha) is evident in both the intestine of IBD mice [45] and obesity-inflamed adipose [46]. Given these differences, we predict that (1) different mechanisms mediate the inflammation of PVAT vs. intestinal inflammation in IBD and (2) adipose inflammation related to obesity likely occurs via unique pathways compared to adipose inflammation in IBD. Given the lack of weight gain in both humans and mice with IBD and the hyperplasia vs. hypertrophy of PVAT adipocytes, the significant PVAT obesity literature may not be relevant when interpreting findings from other disease models such as IBD.

To gain additional insights into mechanisms underlying changes in cytokine and chemokine gene expression, we conducted pathway and reactome analysis using all PCR array genes with fold changes > 1.5 or *p*-value < 0.5. Transcription factor analysis using the TTRUST database highlighted the greatest enrichment of NF-κB pathways (Figure 4A). This is consistent with a known role for NF-κB in initiating adipocyte inflammatory processes, particularly in white adipose [47] as found in mesenteric PVAT. The additional enrichment of RELA and RELB suggest that both canonical and non-canonical pathways may be involved. On the canonical side, increased PCR array expression of the NF-κB activators tumor necrosis factor (TNF) and a number of NF-κB dependent chemokines including genes for IL-6, CCL5 and IL1-β support activation via this mechanism. There is also strong evidence for non-canonical activation with increased expression of the activators *lta* and *ltb* and increased expression of genes for the known downstream chemokine *cxcl13* that is linked to non-canonical NF-κB activation in human IBD patients [48]. Follow-up studies of PVAT function are needed to better elucidate the specific roles of each NF-κB pathway and protein on vasomotor function. Transcriptome analysis of disease associations indicated that our enriched PCR gene sets were most highly associated with ulcerative colitis (Figure 4B), suggesting that our mouse model is accurately recapitulating key aspects of human disease. Finally, the CellMarker database analysis of PCR array genes indicated the greatest enrichment for M1 macrophages in mouse adipose. This is consistent with our finding of increased F4/80+ macrophages in mesenteric PVAT with IBD (Figure 3A,C) and highlights the need for additional future studies looking at the role of macrophage infiltration in PVAT dysfunction during IBD.

This study represents an important addition to our previous work highlighting the inhibitory of adventitial macrophage infiltration on sensory vasodilation [12,13]. Those studies were conducted in arteries devoid of PVAT and therefore did not address its role in either macrophage infiltration or vasomotor function. The major goals of this new study were to determine whether PVAT’s influence on mesenteric artery vasomotor function changes in IBD and, if so, to look for preliminary evidence pointing to potential mechanisms. Collectively, the data show that IBD causes functional PVAT pro-contractility and structural PVAT remodeling that occur in parallel with widespread changes in cytokine and chemokine gene expression. While these novel findings are significant, there are also limitations to the studies that will need to be addressed in future work. First, the macrophage population has not been characterized beyond the broad F4/80 marker. Second, the PCR arrays measured the expression of a subset of cytokine and chemokine genes rather than a full RNA sequencing study of the entire genome. Nevertheless, these data highlights a key role for macrophage-associated PVAT remodeling and mesenteric PVAT pro-contractility during the pathogenesis of IBD. Understanding the underlying mechanisms and preventing or reversing these changes may represent an important avenue to decrease perivascular inflammation and preserve intestinal perfusion in IBD.

## 4. Materials and Methods

### 4.1. Animals and Tissue Collection

Male and female IL10^−/−^ and C57BL/6J mice (Jackson Laboratory B6.129P2-IL-10^tm1Cgn^/J, Strain #002251 & C57BL/6J, Strain #000664) were bred and housed at the University of Missouri in accordance with the Guide for the Care and Use of Laboratory Animals [49] approved by the University of Missouri Animal Care and Use Committee protocol #44228. For all experiments, IBD groups (IL10^−/−^ mice) were orally gavaged with *H. hepaticus* at days 2 and 4 post-weaning as previously described [12,13] with Control groups including age-matched C57BL/6J controls. IBD mice were allowed to develop disease for 90 days post-gavage prior to experimentation. For all experiments, mice were anesthetized via an intraperitoneal injection of ketamine–xylazine (25 mg/mL/2.5 mg/mL) followed by tissue dissection and euthanasia via cardiac exsanguination. The small intestine was placed in 4 °C physiological salt solution (PSS, in mM: 140 NaCl, 5 KCl, 1 MgCl_2_, 10 HEPES, 10 glucose) for the hand dissection of arteries and PVAT under a stereomicroscope.

### 4.2. Isolated Artery Studies

Unbranched 1st or 2nd order MAs with or without attached PVAT were transferred to a cannulation chamber with platinum electrodes (Warner Instruments, Hamden, CT, USA), cannulated onto glass pipettes, and tied with 11–0 sutures as previously described [12,13]. For MAs +PVAT, a small opening was dissected in the PVAT to allow for diameter tracking during myography. All arteries were pressurized to 70 mmHg, heated to 37 °C and superfused with PSS with 2 mM calcium. Images were acquired on an inverted Olympus BX61W1 microscope and a CCD camera (Basler, Inc., Exton, PA, USA) coupled to edge-tracking software (LabView, v2017.11.20, National Instruments, Austin, TX, USA) customized by Dr. Michael J Davis. Preparations were stimulated in an electrical field as previously described [12,50]. Briefly, electrical pulses (70 V, 2 ms) were delivered using a Grass S88 stimulator and stimulus isolation unit at 1–16 Hz until a stable level of vasoconstriction was achieved, typically in 15–30 s.

### 4.3. PVAT Sectioning and Adipocyte Measurements

For adipocyte measurements of PVAT, first-order MAs with surrounding PVAT were hand dissected from Control and IBD mice and fixed in 10% neutral-buffered formalin. Tissue was held in place with two layers of biopsy foam pads within a tissue cassette. At the University of Missouri Veterinary Medical Diagnostic Laboratory, 10 μm sections of each sample were mounted to slides and stained with hematoxylin and eosin. Prepared slides were imaged with a 20× objective on a Nikon E800 microscope with Nikon Elements software (version 5.21). The resulting images were analyzed using the Adiposoft plugin for ImageJ [51], which allowed for the automatic counting and measuring of individual adipocytes within the field of view of each image. After automatic counting was performed by Adiposoft, manual counting by a lab member was performed on a subset of images to validate the accuracy of the adipocyte counts. In both the automated and manual counts, partial cells at the periphery of each image were excluded from analysis. Representative images were selected from both Control and IBD tissues in order to effectively show the size and density differences between the two groups. Nested *t*-tests were performed to compare group means.

### 4.4. Immunofluorescence and Quantitation

First-order MAs with attached PVAT were pinned using a 50 μm wire in a 24-well plate coated with Sylgard. The tissue was fixed in 4% paraformaldehyde for 20 min, blocked with 1% bovine serum albumin, permeabilized for 60 min with 0.1% Triton X-100 and incubated overnight in primary antibody for CD45 to label leukocytes and F4/80 to label macrophages. Tissues were then blocked again for 60 min, incubated in secondary antibodies for 90 min, and mounted on slides. Control experiments included secondary-only and antibody-free controls to account for non-specific labeling and autofluorescence. Slides were imaged using a Leica TCS SP8 confocal laser-scanning microscope (Leica Microsystems). The fluorescence for each antibody was sequentially imaged at 1024 × 1024 pixels using a 25× water immersion objective (NA = 0.95) and 1 μm Z-slices through the tissue samples. Similar laser power and gain settings were used on both the control and IBD arteries to facilitate comparison. CD45 and F4/80 fluorescence was quantified by measuring the fluorescence area of each label in maximum z-projections using ImageJ. Maximum-intensity z-projections were created separately for each channel and converted to binary images with thresholds set to eliminate background fluorescence. First, the maximum z-projections of each channel were exported from LASX and imported to Image J. Background was adjusted to remove non-specific fluorescence. Specifically, the black level (minimum) was increased from 0 to ~30 (on the 0–255 gray-level scale) to remove the low-level adipocyte autofluorescence without eliminating the much brighter signal from labeled macrophages. Binary images were generated from these adjusted images. From the binary image, the ImageJ “measure” feature was used to determine the percent fluorescent area, which was the total fluorescent area fraction within the entire image. Images that could not be adequately adjusted were excluded from analysis. Images were excluded from analysis due to uncorrectable autofluorescent background, immunofluorescent non-biological fibers or dust on tissue samples, or poor image quality.

### 4.5. PCR Arrays

The PVAT surrounding all first- and second-order mesenteric arteries from two mice were combined per sample. Tissue samples were minced with a scalpel and homogenized with a pellet pestle motor homogenizer. RNA was extracted per the kit instruction from PVAT samples using the Macherey-Nagel NucleoSpin RNA kit (740955.50), while the Macherey-Nagel NucleoSpin RNA XS kit (740902.50) was used for MA samples. An on-column DNase digestion step was included, and RNA purity was assed using a NanoDrop Spectrophotometer (Thermo Scientific, Waltham, MA, USA). Due to the low amount of RNA, cDNA was made using the RT^2^ PreAMP cDNA synthesis kit (Qiagen, Venlo, The Netherlands, #330451) and amplified using the RT^2^ PreAMP Pathway Primer Mix (Qiagen, 330241) per kit protocol. cDNA samples were then frozen at −20 °C. The RT^2^ Profiler PCR Array Mouse Cytokines & Chemokines (Qiagen, PAMM-150Z) was run using RT^2^ SYBR Green qPCR MasterMix (Qiagen, #330502) per kit protocol on a CFX96 Touch Real-Time PCR machine (Bio-Rad, San Francisco, CA, USA). Data were uploaded to the GeneGlobe (Qiagen) website for analysis. WT samples were assigned as the control group for analysis. Data quality was assessed by checking the PCR array reproducibility, reverse transcription control (RTC), and genomic DNA contamination (GDC) of all samples. Data are expressed as fold regulation. Fold-change values greater than one indicate an upregulation, and fold-change values less than one indicate a downregulation. The *p*-value for each gene was calculated based on a Student’s *t*-test of expression in Control vs. IBD samples with *p* values less than 0.05 being considered significant. To garner additional functional insights based on PCR array results, we performed additional enrichment analysis of all genes from the PCR arrays with a fold change of >1.5 in IBD vs. Control to focus on pathways upregulated in IBD. Enrichment analyses for transcription factors (TRRUST database [52]), disease associations (DisGeNET database [53]) and cell types (CellMarker 2.0 database [54]) were analyzed using the Enrichr program [55]. Inputs included genes with increased expression in IBD vs. Control from PCR arrays (fold changes > 1.5, adjusted *p* < 0.05).

## Figures and Tables

**Table 1:** Upregulated genes in inflammatory bowel disease (IBD) vs. Control perivascular adipose tissue (PVAT) samples based on Qiagen cytokines and chemokines PCR arrays. Data are mean ± SD C_t_ with associated fold change and adjusted *p*-values. *n* = 4 mice per group. Red = *p* < 0.05. Bold = fold change > 2.0.

Gene	Description	Control		IBD		Fold Change	*p*-Value
		C_t_	SD	C_t_	SD		
**Ltb**	**Lymphotoxin B**	**26.44**	**0.58**	**23.84**	**0.36**	**3.93**	**0.05**
**Ccl12**	**Chemokine (C-C motif) ligand 12**	**28.4**	**1.12**	**26.07**	**0.52**	**3.27**	**0.05**
**Lta**	**Lymphotoxin A**	**30.9**	**0.23**	**28.67**	**0.25**	**3.04**	**0.14**
**Cxcl13**	**Chemokine (C-X-C motif) ligand 13**	**22.94**	**0.78**	**20.84**	**0.35**	**2.8**	**0.04**
**Cxcl1**	**Chemokine (C-X-C motif) ligand 1**	**31.82**	**1.34**	**29.79**	**0.32**	**2.66**	**0.13**
**Ccl5**	**Chemokine (C-C motif) ligand 5**	**25.52**	**0.54**	**23.55**	**0.45**	**2.56**	**0.07**
**Cxcl16**	**Chemokine (C-X-C motif) ligand 16**	**27.26**	**0.8**	**25.55**	**0.41**	**2.12**	**0.05**
Il16	Interleukin 16	26.9	0.65	25.43	0.39	1.79	0.15
Il6	Interleukin 6	31.08	0.1	29.64	0.28	1.76	0.96
B2m	Beta-2 microglobulin	18.54	0.49	17.23	0.57	1.6	0.17
Il17a	Interleukin 17A	32.84	0.23	31.54	0.15	1.6	0.6
Il1b	Interleukin 1 beta	28.37	0.64	27.13	0.28	1.53	0.34
Tnf	Tumor necrosis factor	29.03	0.1	27.82	0.28	1.51	0.98
Ccl1	Chemokine (C-C motif) ligand 1	32.95	0.33	31.79	0.48	1.45	0.42
Ppbp	Pro-platelet basic protein	27.67	0.38	26.52	0.7	1.45	0.86
Ifna2	Interferon alpha 2	30.86	1.66	29.77	0.74	1.38	0.34
Ccl7	Chemokine (C-C motif) ligand 7	25.29	1.13	24.22	0.7	1.36	0.7
Ccl22	Chemokine (C-C motif) ligand 22	29.61	0.74	28.55	0.53	1.36	0.94
Tgfb2	Transforming growth factor, beta 2	27.5	0.95	26.45	0.49	1.35	0.32
Il4	Interleukin 4	29.48	0.36	28.43	0.47	1.35	0.62
Bmp2	Bone morphogenetic protein 2	28.69	0.82	27.67	0.71	1.32	0.36
Fasl	Fas ligand (TNF superfamily, member 6)	29.78	0.21	28.77	0.13	1.31	0.91
Gpi1	Glucose phosphate isomerase 1	21.27	0.74	20.27	0.33	1.3	0.25
Cxcl5	Chemokine (C-X-C motif) ligand 5	30.24	0.33	29.27	0.59	1.27	0.53
Pf4	Platelet factor 4	23.67	0.84	22.71	0.68	1.26	0.27
Nodal	Nodal	30.87	0.61	29.94	0.4	1.24	0.55
Tnfsf13b	Tumor necrosis factor superfamily, 13b	26.59	0.89	25.67	0.42	1.23	0.38
Il21	Interleukin 21	30.63	0.54	29.71	0.25	1.23	0.94
Tnfsf10	Tumor necrosis factor superfamily, member 10	25.92	0.33	25.02	0.13	1.22	0.95
Il11	Interleukin 11	30.51	0.86	29.61	0.1	1.2	0.71
Spp1	Secreted phosphoprotein 1	29.3	0.51	28.41	0.53	1.2	0.83
Tnfsf11	Tumor necrosis factor superfamily, member 11	28.54	0.25	27.67	0.45	1.19	0.82
Gapdh	Glyceraldehyde-3-phosphate dehydrogenase	19.74	0.78	18.88	0.57	1.18	0.28
Cxcl10	Chemokine (C-X-C motif) ligand 10	24.81	0.66	23.96	0.39	1.18	0.4
Ccl2	Chemokine (C-C motif) ligand 2	25.52	0.91	24.68	0.71	1.16	0.53
Il18	Interleukin 18	25.58	1	24.74	0.47	1.16	0.8
Mif	Macrophage migration inhibitory factor	21.88	0.69	21.06	0.44	1.15	0.29
Ccl19	Chemokine (C-C motif) ligand 19	24.28	0.47	23.47	0.25	1.14	0.73
Ifng	Interferon gamma	28.7	0.36	27.91	0.39	1.12	0.93
Ccl24	Chemokine (C-C motif) ligand 24	29.39	1.24	28.62	0.83	1.11	0.96
Cd40lg	CD40 ligand	28.78	0.24	28.02	0.39	1.1	0.66
Adipoq	Adiponectin, C1Q and collagen domain containing	19.16	0.7	18.42	0.7	1.09	0.62
Tnfrsf11b	Tumor necrosis factor receptor superfamily, member 11b	25.67	1.11	24.96	0.7	1.07	0.9
Xcl1	Chemokine (C motif) ligand 1	27.99	0.32	27.28	0.35	1.06	0.69
Il1rn	Interleukin 1 receptor antagonist	28.55	0.44	27.85	0.32	1.05	0.9
Hsp90ab1	Heat shock protein 90 alpha, class B member 1	19.29	0.76	18.62	0.41	1.03	0.66
Ccl11	Chemokine (C-C motif) ligand 11	24.31	0.69	23.67	0.68	1.01	0.77

**Table 2:** Downreguated genes in IBD vs. Control PVAT samples based on Qiagen cytokines and chemokines PCR arrays. Data are mean ± SD C_t_ with associated fold change and adjusted *p*-values. *n* = 4 mice per group. Red = *p* < 0.05. Bold = fold change < 0.5.

Gene	Description	Control		IBD		Fold Change	*p*-Value
		C_t_	SD	C_t_	SD		
**Il13**	**Interleukin 13**	**28.75**	**0.24**	**29.39**	**0.47**	**0.42**	**0.17**
**Il24**	**Interleukin 24**	**28.87**	**0.11**	**29.38**	**0.32**	**0.46**	**0.16**
**Csf3**	**Colony stimulating factor 3**	**29.43**	**0.28**	**30.89**	**0.58**	**0.37**	**0.05**
**Ccl3**	**Chemokine (C-C motif) ligand 3**	**28.61**	**0.67**	**29**	**0.5**	**0.5**	**0.02**
**Il22**	**Interleukin 22**	**28.63**	**0.33**	**28.96**	**0.46**	**0.5**	**0.13**
**Csf2**	**Colony stimulating factor 2**	**30.07**	**0.45**	**30.39**	**0.37**	**0.5**	**0.16**
**Cd70**	**CD70 antigen**	**29.76**	**0.44**	**30.08**	**0.87**	**0.5**	**0.22**
Il2	Interleukin 2	29.88	0.15	30.13	0.41	0.55	0.64
Hc	Hemolytic complement	29.02	0.39	29.2	0.34	0.57	0.14
Cxcl3	Chemokine (C-X-C motif) ligand 3	29.98	0.25	30.14	0.3	0.58	0.28
Il3	Interleukin 3	29.56	0.2	29.67	0.32	0.6	0.29
Mstn	Myostatin	29.57	0.35	29.66	0.36	0.61	0.19
Il23a	Interleukin 23, alpha subunit p19	28.97	0.36	28.97	0.51	0.65	0.22
Cxcl11	Chemokine (C-X-C motif) ligand 11	26.78	0.46	26.73	0.42	0.67	0.22
**Thpo**	**Thrombopoietin**	**27.29**	**0.71**	**27.18**	**0.51**	**0.7**	**0.01**
Cntf	Ciliary neurotrophic factor	27.84	0.58	27.72	0.41	0.71	0.39
Il5	Interleukin 5	28.99	0.33	28.84	0.44	0.73	0.29
Cxcl12	Chemokine (C-X-C motif) ligand 12	22.05	0.72	21.85	0.38	0.75	0.21
Il1a	Interleukin 1 alpha	28.76	0.41	28.56	0.29	0.75	0.54
Bmp4	Bone morphogenetic protein 4	25.29	0.93	25.07	0.58	0.76	0.36
Il7	Interleukin 7	26.21	0.77	25.96	0.46	0.77	0.14
Il17f	Interleukin 17F	29.58	0.37	29.35	0.45	0.77	0.25
Ccl20	Chemokine (C-C motif) ligand 20	31.2	0.15	30.94	0.38	0.78	0.41
Bmp6	Bone morphogenetic protein 6	24.81	0.63	24.53	0.26	0.79	0.5
Il15	Interleukin 15	25.48	0.64	25.17	0.34	0.8	0.31
Vegfa	Vascular endothelial growth factor A	20.95	0.71	20.63	0.37	0.81	0.36
**Csf1**	**Colony stimulating factor 1**	**23.06**	**0.79**	**22.7**	**0.48**	**0.83**	**0.04**
Bmp7	Bone morphogenetic protein 7	27.18	0.86	26.8	0.5	0.84	0.33
Ctf1	Cardiotrophin 1	26.71	0.75	26.32	0.49	0.85	0.25
Il12a	Interleukin 12A	28.66	0.38	28.28	0.31	0.85	0.48
Il9	Interleukin 9	29.66	0.35	29.25	0.27	0.86	0.61
Ccl17	Chemokine (C-C motif) ligand 17	28.44	0.29	28.02	0.32	0.87	0.46
Cx3cl1	Chemokine (C-X3-C motif) ligand 1	27.4	0.69	26.94	0.39	0.89	0.56
Ccl4	Chemokine (C-C motif) ligand 4	28.24	0.54	27.79	0.35	0.89	0.74
Cxcl9	Chemokine (C-X-C motif) ligand 9	27.7	0.12	27.21	0.54	0.92	0.43
Osm	Oncostatin M	28.93	0.51	28.43	0.46	0.92	0.52
Lif	Leukemia inhibitory factor	29.05	0.61	28.5	0.23	0.95	0.99
Il12b	Interleukin 12b	28.63	0.3	28.06	0.32	0.96	0.76
Gusb	Glucuronidase, beta	23.11	0.74	22.52	0.54	0.97	0.8
Il27	Interleukin 27	29.31	0.44	28.7	0.65	0.99	0.65

## Data Availability

The original contributions presented in the study are included in the article. Further inquiries can be directed to the corresponding author.
